# Mild and efficient cyanuric chloride catalyzed Pictet–Spengler reaction

**DOI:** 10.3762/bjoc.9.140

**Published:** 2013-06-26

**Authors:** Ashish Sharma, Mrityunjay Singh, Nitya Nand Rai, Devesh Sawant

**Affiliations:** 1 Department of Medicinal Chemistry, National Institute of Pharmaceutical Education and Research (NIPER)-Rae Bareli, ITI Compound, Rae Bareli-229010 (UP), India

**Keywords:** β-carboline, cyanuric chloride, 6-*endo* cyclization, Pictet–Spengler, TCT

## Abstract

A practical, mild and efficient protocol for the Pictet–Spengler reaction catalyzed by cyanuric chloride (trichloro-1,3,5-triazine, TCT) is described. The 6-*endo* cyclization of tryptophan/tryptamine and modified Pictet–Spengler substrates with both electron-withdrawing and electron-donating aldehydes was carried out by using a catalytic amount of TCT (10 mol %) in DMSO under a nitrogen atmosphere. TCT catalyzed the Pictet–Spengler reaction involving electron-donating aldehydes in excellent yield. Thus, it has a distinct advantage over the existing methodologies where electron-donating aldehydes failed to undergo 6-*endo* cyclization. Our methodology provided broad substrate scope and diversity. This is indeed the first report of the use of TCT as a catalyst for the Pictet–Spengler reaction.

## Introduction

The Pictet–Spengler reaction is an important class of name reaction employed extensively for the synthesis of tetrahydro-β-carboline [1–9]. Typically, the Pictet–Spengler reaction is a two-step reaction. The first step is the condensation of aliphatic amine substrates such as tryptophan/tryptamine and aldehydes to generate the intermediate imine in situ. The intermediate imine then undergoes a 6-*endo* cyclization to furnish the cyclized product, tetrahydro-β-carboline [10]. Recently, arylamines have been employed instead of the aliphatic amines for the Pictet–Spengler reaction. These reactions are generally termed as modified Pictet–Spengler reaction [11]. This modification of the conventional reaction led to the synthesis of a polyheterocyclic skeleton that mimics the natural products [12–17]. The 6-*endo* cyclization is the rate-limiting step in the Pictet–Spengler reaction and electrophilicity of the imine is the driving force of the reaction [1]. The Pictet–Spengler reaction is catalyzed by Brønsted acids, as they convert the intermediate imine to the corresponding iminium ion, thus making it more electrophilic [18–22]. Conventionally, TFA [18], HCl [20], H_2_SO_4_ [21] and *p*-TsOH [22] are employed as Brønsted acids. However, recently several other aprotic or Lewis acids, such as Yb(OTf)_3_ [23–25], AuCl_3_/AgOTf [26], Me_3_SiCl [27–28], BF_3_·Et_2_O [29], iodine [30], zeolite [31] and enzymes [32–34], have been used for carrying out the Pictet–Spengler reaction.

Though the Pictet–Spengler reaction has been studied extensively under a variety of Brønsted/Lewis acid catalyzed conditions [35], most of these conditions failed to facilitate a 6-*endo* cyclization where salicylaldehyde (or any electron-donating aldehyde such as 4-dimethylaminobenzaldehyde) was used as a source of aldehyde [1,22]. Even harsher reaction conditions such as heating at a high temperature furnished the completely oxidized product, β-carboline [1]. The reason for this failure could be attributed to the reduced electrophilicity of the corresponding imine generated from these aldehydes. As a result of its reduced electrophilicity the imine failed to cyclize under the conditions reported earlier. A literature search revealed that the acyliminium strategy has been used to overcome this defect [25,36–37]. However, one has to cleave the *N*-acyl group of the product in order to generate the desired compound, and thus, an additional deprotection step has to be introduced. Instead, a mild and efficient catalyst that can effectively produce iminium species in situ could generate the compound of interest without adding any extra steps. We envisaged that the use of cyanuric chloride (2,4,6-trichloro-1,3,5-triazine, TCT) [38–45] as a source of HCl could trigger the 6-*endo* cyclization under dry and mild conditions. TCT is used in many reactions as a source of anhydrous HCl in organic synthesis [46]. Moreover, it is an attractive reagent due to its easy availability and low cost. It is well documented in the literature that TCT reacts with the incipient moisture and produces three moles of HCl and cyanuric acid as a byproduct [46]. The latter can be removed by a simple aqueous workup. Interestingly, this reagent has not been explored for the Pictet–Spengler reaction yet. Here we report the use of cyanuric chloride as a simple, inexpensive and efficient catalyst for the Pictet–Spengler reaction.

## Results and Discussion

**Scheme 1:**
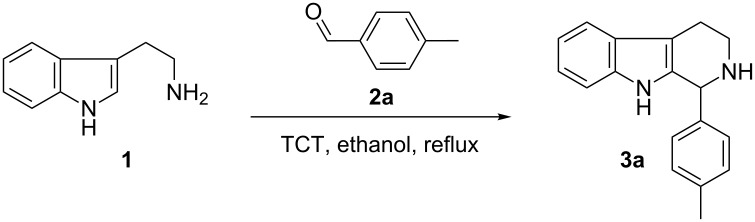
The Pictet–Spengler reaction of tryptamine with 4-tolualdehyde.

We began our studies by reacting tryptamine (**1**) and 4-tolualdehyde (**2a**) in the presence of 20 mol % TCT in ethanol (Scheme 1). The reaction showed the formation of the cyclized product **3a** albeit with the presence of starting materials even after prolonged heating (16 h). Encouraged by this initial observation, we focused our attention on optimizing the reaction conditions. First, various solvents were examined. These findings are reported in Table 1. In general, the reaction proceeded well in polar solvents such as EtOH, acetonitrile and DMSO. In contrast, the reaction failed to produce the desired compounds, when nonpolar solvents such as toluene, DCM, dioxane and THF were used [47]. DMSO produced the best results, even though fully aromatized β-carboline was obtained as a side product. This drawback can be excluded by employing an inert atmosphere. Thus, reactions carried out in DMSO at 100 °C by using 20 mol % TCT under nitrogen (Table 1, entry 8) demonstrated the best results. Next, we examined the amount of TCT needed for the cyclization and 20 mol % of TCT was found to be optimal for the reaction. An increase of the catalytic loading led to the appearance of side products on a TLC. Reaction temperature was found to be optimal at 100 °C, since a lower temperature (50 to 80 °C) required longer reaction time (48 h) for the completion, and a higher temperature (beyond 120 °C) produced undesired side products [48].

**Table 1:** Method development of the TCT-catalyzed Pictet–Spengler reaction for the condensation of tryptamine (**1**) and 4-tolualdehyde (**2a**).

Entry	Solvent	Temp. (°C)	TCT (mol %)	Time (h)	Isolated yield (%)

1	EtOH	85	20	16	61^a^
2	acetonitrile	85	20	16	75^a^
3	toluene	110	20	16	NR^b^
4	DMSO	100	5	16	45
5	DMSO	100	10	16	68
6	DMSO	100	20	4	85^c^
7	DMSO	100	30	8	78^d^
8	DMSO/N_2_	100	20	8	92

^a^Starting material recovered, ^b^an intermediate imine observed as the major product, ^c^fully aromatic β-carboline observed as a side product on TLC, ^d^formation of multiple spots observed on TLC.

**Figure 1:**
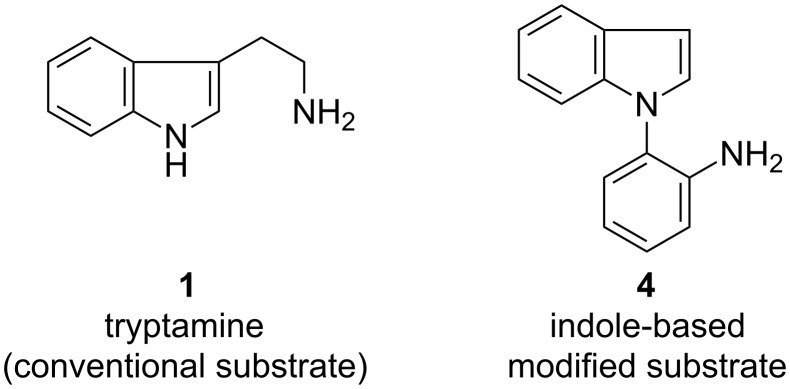
The two Pictet–Spengler substrates employed in the TCT catalyzed cyclization.

**Scheme 2:**
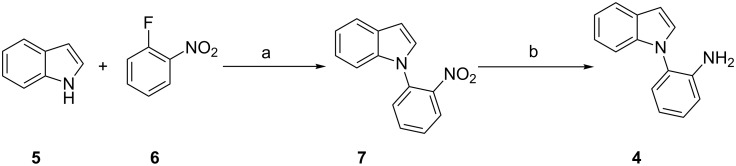
Synthesis of the Pictet–Spengler substrate **4**. Reaction conditions: (a) K_2_CO_3_, DMF, 80 °C, 3 h; (b) Zn, AcOH-EtOH, 60 °C, 4 h.

Once we had identified the optimized reaction conditions, we scrutinized the substrate scope and limitation of the TCT-catalyzed Pictet–Spengler reaction. We explored two different Pictet–Spengler substrates for the TCT-catalyzed reaction as depicted in Figure 1. Tryptamine (**1**) was commercially available, while substrate **4** was synthesized from the nucleophilic substitution of indole (**5**) with 2-fluoronitrobenzene (**6**) to generate intermediate **7**, which was reduced to amine **4** by Zn/HCl (Scheme 2).

Both substrates reacted with benzaldehyde derivatives and results are summarized in Table 2. Tryptamine (**1**) reacted well with aldehydes with both electron-donating and electron-withdrawing substituents to furnish 1,2,3,4-tetrahydro-β-carboline **3**. It is interesting to note that salicylaldehyde (**2g**) and 4-dimethylaminobenzaldehyde (**2h**) produced the desired cyclized products **3g** and **3h**, respectively, when condensed with tryptamine (**1**) in the presence of cyanuric chloride. It is reported in the literature that the condensation of electron-donating aldehydes, such as salicylaldehyde with tryptophan methyl ester or tryptamine in acidic media, provided the 6-*endo* cyclized product in poor yield along with impurities [1,22]. In contrast, our methodology proved effective in catalyzing the condensation of tryptamine and electron-donating aldehydes. Interestingly, the ortho-substitution on benzaldehyde is also well tolerated. For instance, the condensation of 2-bromobenzaldehyde (**2b**) or 2-nitrobenzaldehyde (**2f**) with tryptamine in the presence of TCT furnished the title compounds **3b** or **3f**, respectively, in excellent yield.

**Table 2:** Condensation of the Pictet–Spengler substrates **1** and **4** (1 equiv) with aldehyde (1 equiv) in the presence of TCT (20 mol %) in DMSO under a nitrogen atmosphere.

Entry	Substrate	Aldehyde	Product	Time (h)	Yield (%)	mp (°C)	Lit. mp (°C)	ESI: *m*/*z* (%)

1	**1**	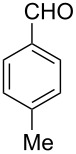 **2a**	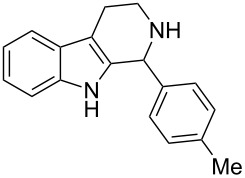 **3a**	3	92	138–139	135–137 [19]	263 [M + H^+^, 100]
2	**1**	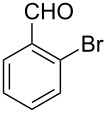 **2b**	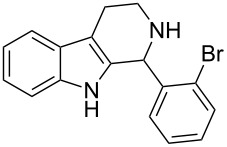 **3b**	4	83	155–157	—^a^ [49]	327 [M + H^+^, 100]; 329 [M + H^+^, 97]
3	**1**	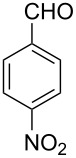 **2c**	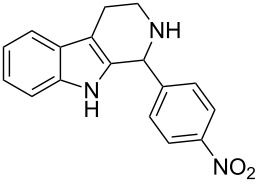 **3c**	2	95	169–171	170–171 [50]	294 [M + H^+^, 100]
4	**1**	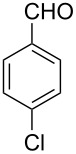 **2d**	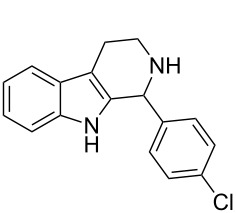 **3d**	5	92	205–206	207–208 [50]	283 [M + H^+^, 100]
5	**1**	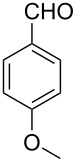 **2e**	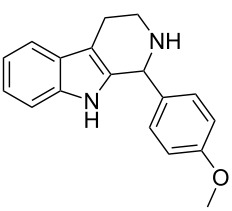 **3e**	6	85	191–193	—^b^ [51]	279 [M + H^+^, 100]
6	**1**	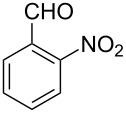 **2f**	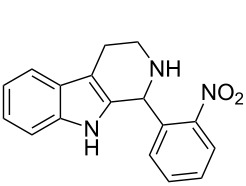 **3f**	3	88	151–152	—^c^ [52]	294 [M + H^+^, 100]
7	**1**	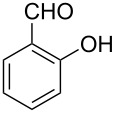 **2g**	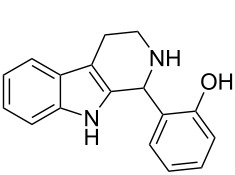 **3g**	4	84	190–191	189–191 [19]	265 [M + H^+^, 100]
8	**1**	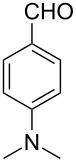 **2h**	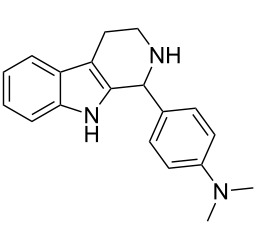 **3h**	6	79	231–233	235–237 [53]	292 [M + H^+^, 100]
9	**4**	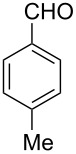 **2a**	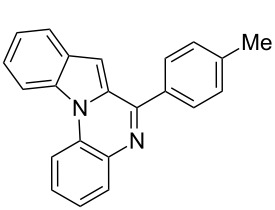 **8a**	2	91	160–162	—	309 [M + H^+^, 100]
10	**4**	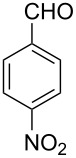 **2c**	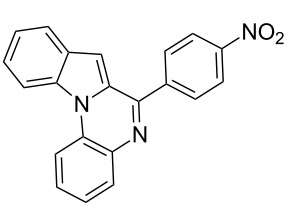 **8b**	1	88	214–215	213–214 [12]	340 [M + H^+^, 100]
11	**4**	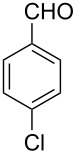 **2d**	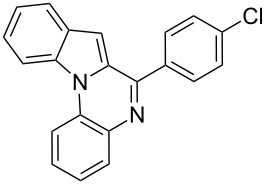 **8c**	2	95	205–207	—	329 [M + H^+^, 100]
12	**4**	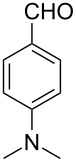 **2h**	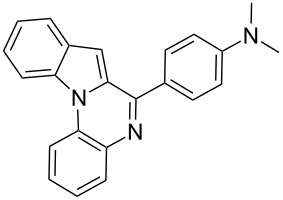 **8d**	3	85	146–147	145–147 [12]	338 [M + H^+^, 100]

^a^No melting point reported, spectral data of **3b** is consistent with the reported spectra. ^b^No melting point reported. Spectral data of **3e** are consistent with the reported spectra. ^c^No physical and spectral data are reported.

The Pictet–Spengler reaction is a two-step process, where an aldehyde and an amine first react to give an imine as an intermediate. This is followed by a 6-*endo* intramolecular cyclization of the imine to produce the cyclized product. The second step is the rate-limiting step and the electrophilicity of the imine is the driving force of the cyclization. HCl generated in situ from TCT protonated the imine intermediate to an iminium ion, which undergoes a cyclization. The presence of an inert nitrogen atmosphere abated formation of the oxidized product (β-carboline), thus allowing for a clean reaction. In a nutshell, the Pictet–Spengler reaction of tryptamine can be elegantly catalyzed by TCT with a high substrate scope and diversity, hence TCT proved to be a robust catalyst in the 6-*endo* cyclization.

Recently, the Pictet–Spengler reaction has been successfully extended to arylamine substrates [11–17]. These substrates are designed by replacing the aliphatic amine side chain attached to activated heterocycles, for example tryptamine (**1**), by aromatic amines [11]. Moreover, the aromatic amines can be originated from either carbon or nitrogen of the activated heterocycle. Hence, these substrates are referred to as “modified Pictet–Spengler substrates”. We employed one of the arylamine substrates for our studies (Figure 1). In substrate **4** (Figure 1), the aromatic amine side chain originates from the *N*-1 of indole. It was subjected to the TCT catalyzed Pictet–Spengler reaction with benzaldehyde derivatives. The results are summarized in Table 2.

The substrate **4** reacted equally well with both electron-donating as well as electron-withdrawing aldehydes to yield the 6-*endo* product indolo[1,2-*a*]quinoxalines **8** (Table 2). It is interesting to note that the cyclized products of the modified Pictet–Spengler substrate **4** are fully aromatized. This is probably because the cyclized dihydro derivatives formed in situ are prone to oxidation. Substrate **4** furnished the cyclized product faster compared to tryptamine (**1**). This might be attributed to the enhanced electrophilicity of the imine derived from the arylamine compared to the aliphatic amine as reported earlier [16].

The TCT catalyzed condensation of the Pictet–Spengler substrates **1** and **4** with aldehydes furnished tetrahydro-β-carboline **3** and indolo[1,2-*a*]quinoxaline **8**, respectively. The β-carboline skeleton is widely distributed in marine organisms [54–56]. Eudistomine and manzamine isolated from marine invertebrates exhibited promising anticancer activity [57–58]. Tedalafil, a drug based on the tetrahydro-β-carboline skeleton, is widely used to treat erectile dysfunction [59]. Hence, these nuclei constitute an important class of heterocyclic compounds, which posses a wide range of pharmacological properties.

## Conclusion

Here we disclosed the first report of the Pictet–Spengler reaction catalyzed by cyanuric acid. Both conventional and modified Pictet–Spengler substrates reacted equally well with electron-donating and electron-withdrawing aldehydes. TCT proved effective in catalyzing the 6-*endo* cyclization of aldehydes such as salicylaldehyde and 4-dimethylaminobenzaldehyde. These aldehydes failed to produce the cyclized product in desired yield under conventional Brønsted acid catalysis and often produced the oxidized products under harsher reaction conditions. TCT under an inert atmosphere allowed for a clean reaction with a broad substrate scope and application. We demonstrated application of this methodology for the synthesis of tetrahydro-β-carbolines **3** and indolo[1,2-*a*]quinoxalines **8**. These scaffolds are present in numerous biologically active compounds. Nevertheless, TCT is inexpensive and readily available. Therefore, this methodology can be easily employed in the synthesis of a spectrum of pharmacologically active compounds on multigram to industrial scales.

## Experimental

A typical experimental procedure: Cyanuric chloride (10 mol %) was added to the mixture of tryptamine (**1**) or arylamine substrate **4** (1 mmol) and aldehyde **2** (1 mmol) in DMSO at rt under a nitrogen atmosphere. The resultant mixture was warmed at 100 °C and stirred for 8 h. The reaction mixture was poured on the bed of crushed ice to obtain the crude. The solid so obtained was filtered and washed with chilled water and 10% EtOAc/hexane solution to remove water-soluble side products and excess aldehydes. Wherever needed the crude was further purified either by recrystallization in EtOH or by flash chromatography.

**1-(4-Methylphenyl)-2,3,4,9-tetrahydro-1*H*-pyrido[3,4-*b*]indole (3a):** Yield 92%; pale-yellow solid; mp 138–139 °C; ^1^H NMR (CDCl_3_, 300 MHz) δ 7.94 (br s, 1H), 7.60–7.57 (m, 1H), 7.18–7.13 (m, 7H), 5.10 (s, 1H), 3.39–3.32 (m, 1H), 3.17–3.08 (m, 1H), 2.99–2.84 (m, 2H), 2.39 (s, 3H); ^13^C NMR (CDCl_3_, 75.5 MHz) δ 138.91, 138.05, 136.00, 134.81, 129.57, 128.57, 127.50, 121.73, 119.42, 118.29, 110.97, 110.15, 57.85, 42.86, 22.63, 21.29, IR (KBr): 3426, 3309, 2924, 2852, 1595 cm^–1^; MS (ES^+^) *m*/*z*: 263.2 [M + H]^+^.

***N*-(4-Indolo[1,2-*a*]quinoxalin-6-ylphenyl)dimethylamine (8d):** yield 85%, yellow solid, mp 149–151 °C; ^1^H NMR (CDCl_3_, 300 MHz) δ 8.52 (d, *J* = 8.2 Hz, 1H), 8.07 (dd, *J* = 1.2, 7.9 Hz, 1H), 8.03 (d, *J* = 8.8 Hz, 2H), 7.96 (d, *J* = 7.8 Hz, 1H), 7.75 (d, *J* = 8.9 Hz, 1H), 7.61–7.54 (m, 2H), 7.45 (t, *J* = 7.4 Hz, 1H), 7.36 (s, 1H), 6.90 (d, *J* = 8.8 Hz, 2H), 6.72 (d, *J* = 8.8 Hz, 1H), 3.10 (s, 6H); ^13^C NMR (75 MHz, CDCl_3_) δ 151.80, 136.67, 133.02, 131.96, 130.11, 129.97, 129.87, 129.36, 129.33, 127.42, 126.10, 124.02, 122.65, 122.44, 114.59, 114.55, 111.85, 110.98, 102.37, 40.52; IR (KBr): 3020, 2924, 1599 cm^–1^; MS (ES^+^) *m*/*z:* 338.3 [M + H]^+^.

## Supporting Information

File 1 Analytical data and copies of ^1^H and ^13^C NMR of **3a**, **3c**, **3h** and **8d**.
