# Supplementary material for: Mild and efficient cyanuric chloride catalyzed Pictet–Spengler reaction
Source: Beilstein J Org Chem. 2013 Jun 26;9:1235–42. doi: 10.3762/bjoc.9.140 (PMC3701375; doi:10.3762/bjoc.9.140)
Supplement: File 1 — Analytical data and copies of 1H and 13C NMR of 3a, 3c, 3h and 8d. [file Beilstein_J_Org_Chem-09-1235-s001.pdf]

Supporting Information

for

Mild and efficient cyanuric chloride catalyzed Pictet–Spengler reaction

Ashish Sharma, Mrityunjay Singh, Nitya Nand Rai and Devesh Sawant*

Address: Department of Medicinal Chemistry, National Institute of Pharmaceutical Education and Research (NIPER)-Rae Bareli, ITI Compound, Rae Bareli-229010 (UP), India

Email: Devesh Sawant - devesh.sawant@niperraebareli.edu.in

* Corresponding author

Analytical data and copies of ^{1}H and ^{13}C NMR of **3a**, **3c**, **3h** and **8d**

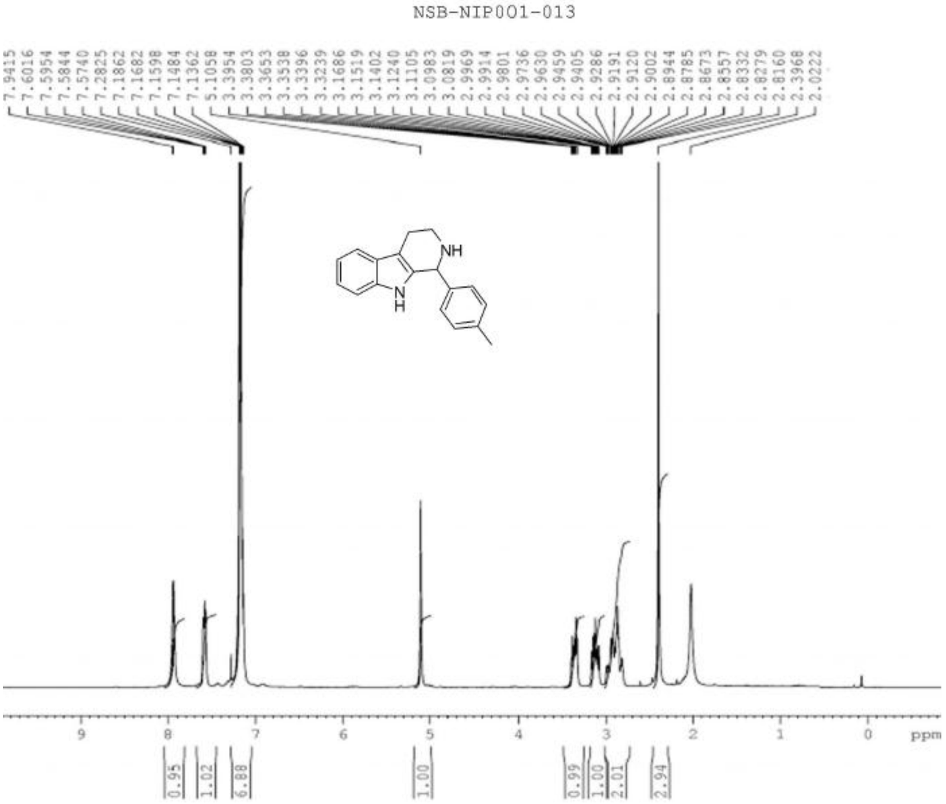

Figure S1: ^{1}H NMR of **3a**.

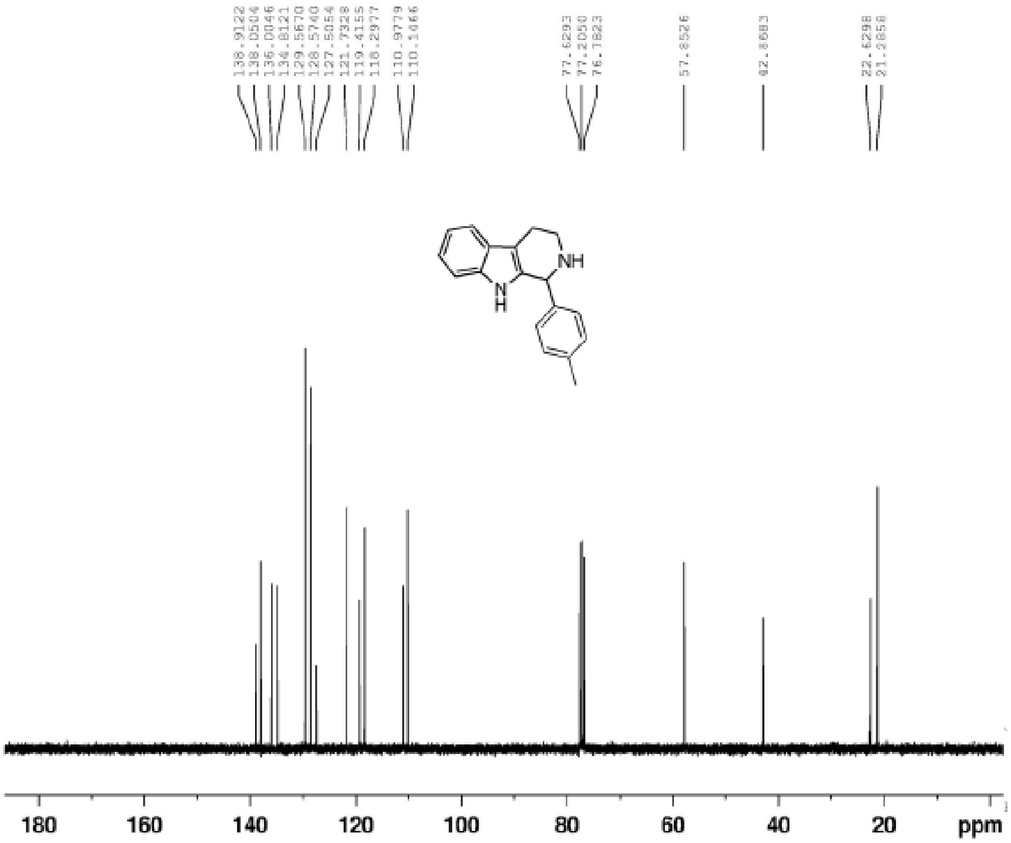

Figure S2: ^{13}C NMR of **3a**.

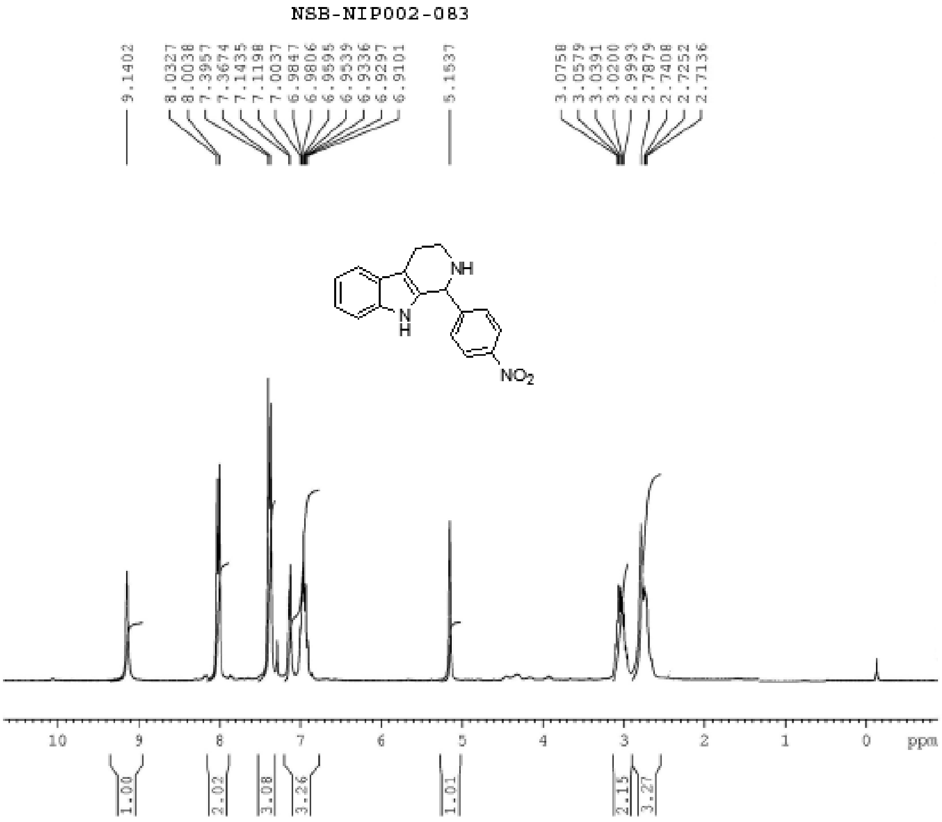

Figure S3: ^{1}H NMR of **3c**.

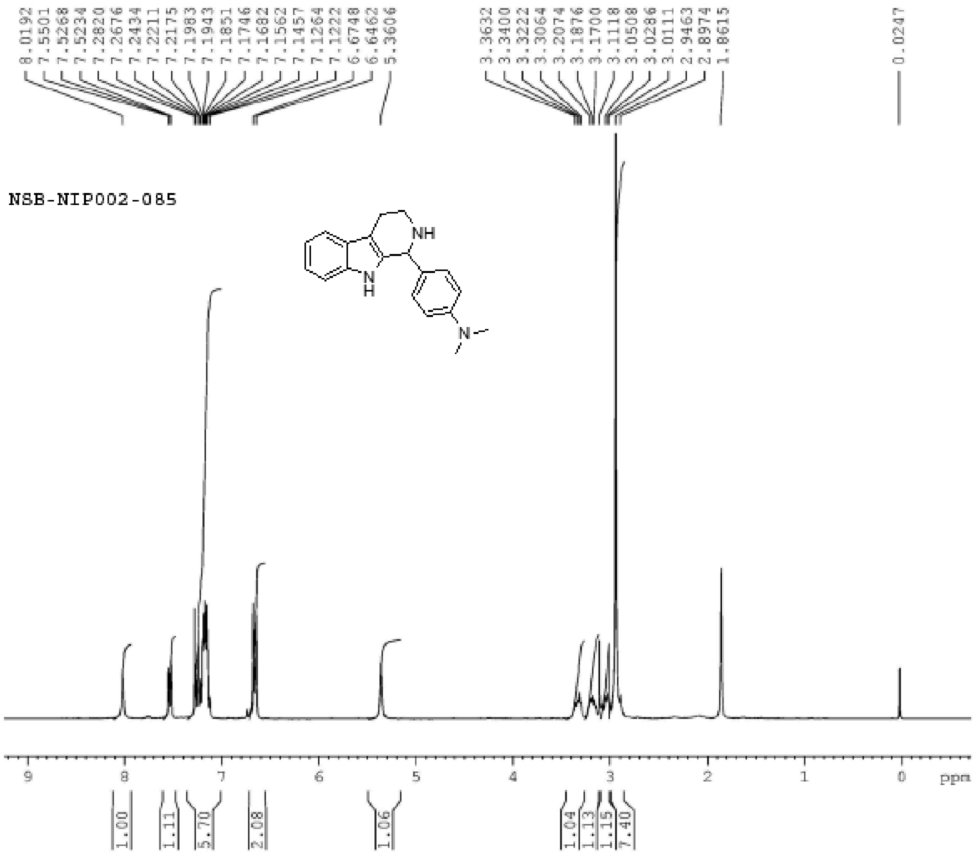

Figure S4: ^{1}H NMR of **3h**.

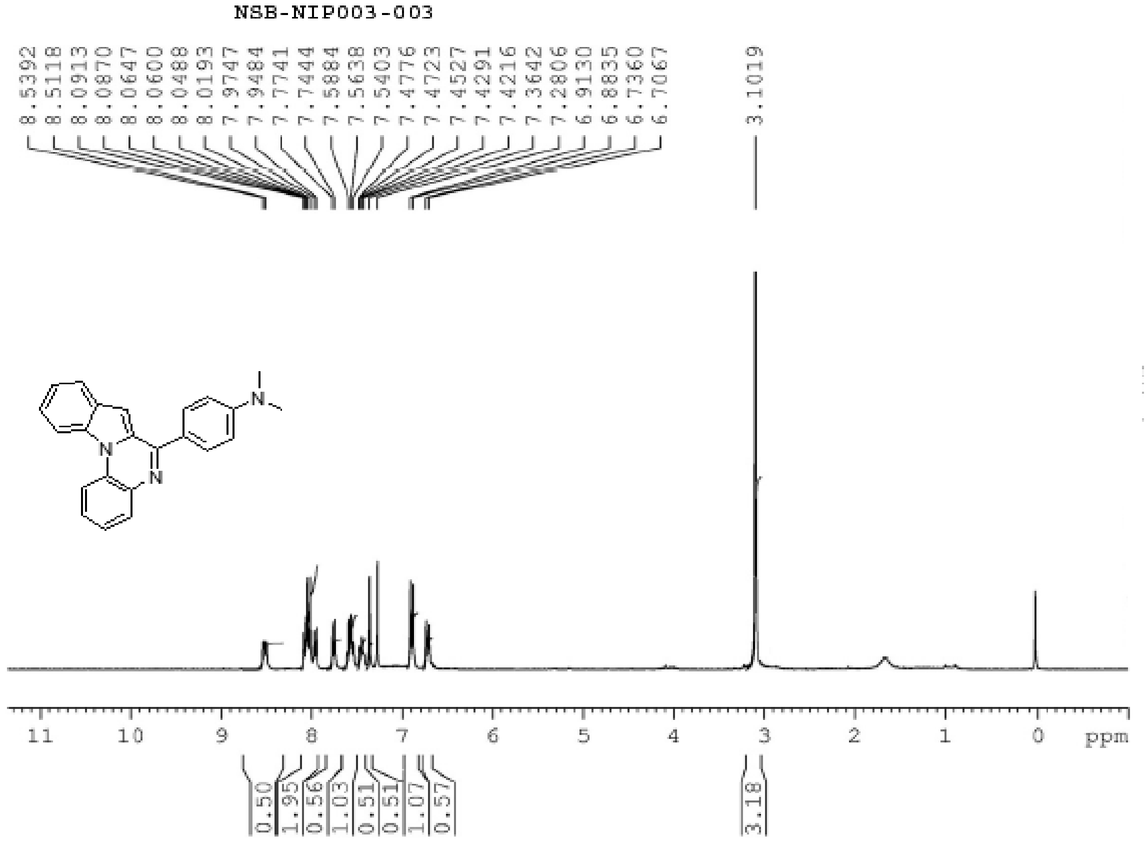

Figure S5: ^{1}H NMR of **8d**.

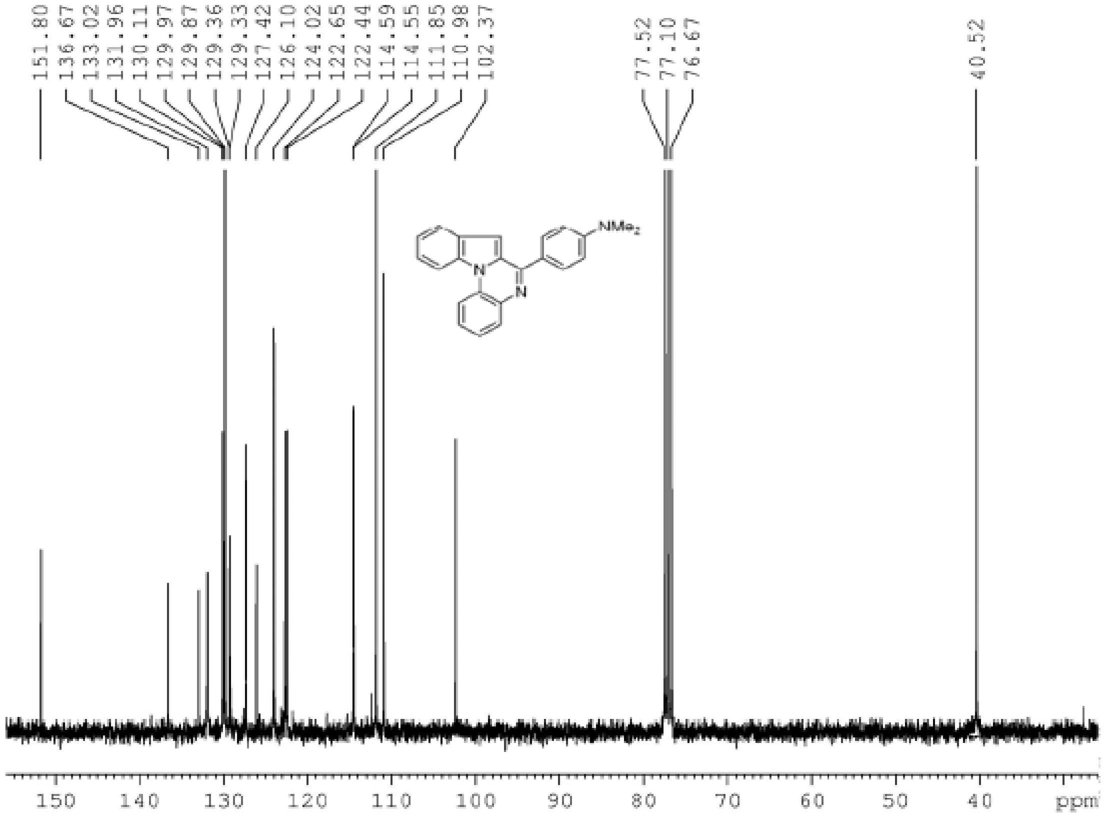

Figure S6: ^{13}C NMR of **8d**.
